# Global, regional, and national burdens of MDR-TB attributable to smoking from 1990 to 2021 with a prediction from 2022 to 2050

**DOI:** 10.3389/fpubh.2025.1634772

**Published:** 2025-09-19

**Authors:** Du Dan, Zhang Lei, Wen Xue, Liu Ze-Xin

**Affiliations:** ^1^Department of Respiratory and Critical Care Medicine, The Affiliated Hospital of Guizhou Medical University, Guiyang, China; ^2^Department of Respiratory and Critical Care Medicine, The Second Hospital of Hebei Medical University, Shijiazhuang, China

**Keywords:** smoking, multidrug-resistant tuberculosis (MDR-TB), mortality, disability-adjusted life years (DALYs), GBD database

## Abstract

**Objective:**

The aim was to offer a comprehensive epidemiological assessment of the global prevalence and the smoking-related Multidrug-resistant tuberculosis (MDR-TB) disease burden from 1990 to 2021 and to forecast the trends in smoking burden over three decades.

**Methods:**

We compared the burden of smoking-related MDR-TB and temporal trends by gender, age, socio-demographic index (SDI), region, and country. Forecasting analyses of the changing trend in the burden of smoking-related MDR-TB up to 2050 was conducted based on the ARIMA model and ES models.

**Results:**

The global age-standardized rate (ASR) of smoking-related MDR-TB increased from 1990 to 2021, highlighting a significant disease burden. In 2021, the cumulative Disability adjusted life years (DALYs) attributed to MDR-TB tallied up to 239,707 cases, with Lesotho, Uzbekistan, Kyrgyzstan, bearing the brunt. The likelihood of developing MDR-TB rose as individuals advanced in years, manifesting most acutely among men aged 35–39 in lower SDI and Low-middle SDI regions. Predictive analysis suggests that by 2050, deaths and DALYs of smoking-related MDR-TB, as well as their corresponding ASR, will continue to decrease.

**Conclusion:**

The burden of MDR-TB worldwide, adjusted for age, and related to smoking, has shown a decline from 1990 to 2021. However, regional disparities have been identified, with some areas experiencing an increase in this burden. These regions with a higher burden emphasize the necessity for the implementation of strong tobacco control measures.

## Introduction

Tuberculosis remains a major public health concern and a danger to global health. Multidrug-resistant tuberculosis (MDR-TB) is characterized by resistance to at least isoniazid and rifampicin (1). The burden of multidrug-resistant tuberculosis has been estimated in 410,000 cases in 2022 (2). In worldwide, MDR-TB affects 3.3% of individuals newly diagnosed with tuberculosis (TB) and is found in 18% of those with a history of prior TB treatment (3). Elevated rates of MDR-TB are particularly evident in Russia, as well as in several countries throughout Asia and Eastern Europe (4, 5). China ranks among the top seven nations globally in terms of the burden of MDR-TB, accounting for approximately two-thirds of MDR/RR-TB cases worldwide (6). Epidemiological studies indicate a clear association between MDR-TB and age, with young adults-especially those between 26 and 45 years old-showing increased susceptibility (7). Management of MDR-TB remains particularly challenging owing to complex drug regimens, side effects of second-line agents, and substantial treatment expenses. Therefore, understanding the risk factors associated with MDR-TB is of critical importance.

We know for instance that smoking is the leading risk factor for increased mortality among males (and risk of TB infection, active TB disease, and TB mortality when co-existing with TB) (8, 9). This is especially important among Asian and African countries with high rates of TB and smoking (10). Currently 80% of smokers reside in LMICs—where we see the highest rates of TB. There will continue to be promotions of cigarettes through various channels with for instance BAT generating sales of US$34billion in 2022 and Philip Morris US$31.8 billion (11).

The Global Adult Tobacco Survey (GATS) found that tobacco-related diseases claim over 7 million lives globally every year (12). There is considerable evidence indicating a link between tobacco smoking and TB infection, with smokers exhibiting twice the risk of TB infection and progression to active TB (13). Furthermore, cigarette smoking is not only strongly correlated with a heightened risk of TB but is also linked to the recurrence and severity of pulmonary TB (14, 15). The finding that the risk of TB could be reduced by nearly two-thirds upon quitting smoking underscores the critical role of smoking in the fight against TB (16). Nevertheless, many relevant studies were constrained by limited sample sizes and relied more on case–control designs rather than cohort studies. Moreover, the epidemiological evidence regarding this relationship has yet to be comprehensively synthesized.

Following inhalation, *Mycobacterium tuberculosis* mainly targets and multiplies inside alveolar macrophages (AMs). Exposure to tobacco smoke, however, triggers substantial phenotypic and metabolic changes in these immune cells, reducing their ability to phagocytose and weakening their anti-mycobacterial activity (17, 18). Moreover, smoking undermines host immunity through several mechanisms: (1) impairing mucociliary clearance, (2) inhibiting the secretion of TNF-α and IL-12, and (3) disturbing the process of granuloma formation (19–21). It is plausible that smoking-related systemic immunosuppression may facilitate infection with drug-resistant TB strains, including MDR-TB. Additionally, TB patients who smoke demonstrated poorer adherence to treatment and a reduced tendency to complete therapeutic regimens, thereby increasing the risk of acquired drug resistance (22, 23). Multivariate logistic regression analysis showed that the risk of developing MDR-TB in individuals with comorbidities was 9.17 times higher than in individuals without comorbidities (24).

While prior Global Burden of Disease (GBD) studies have identified smoking as a risk factor for MDR-TB, they have not fully assessed the overall burden attributable to smoking in MDR-TB cases. Therefore, mapping the global distribution of smoking-related MDR-TB burden is critical. Using publicly available GBD data, we quantify its evolving impact over three decades (1990–2021).

## Methods

### Study data

We sourced MDR-TB data from the Global Burden of Disease 2021 database (GBD 2021; http://ghdx.healthdata.org/gbd-2021), a comprehensive epidemiological resource spanning 204 countries and territories. The GBD 2021 covers 369 diseases and 87 risk factors, with nations grouped into 21 standardized regions including Sub-Saharan Africa, High-Income North America, and Asia-Pacific (25, 26). The foundational data on the smoking-related burden of MDR-TB for this analysis, comprising estimated deaths, DALYs, and ASR, were sourced from the Global Health Data Exchange query tool(accessed October 10 2024) (27). Our study follows GATHER guidelines and Declaration of Helsinki principles (28), with ethical approval from UW IRB (STUDY00009060). Only de-identified GBD data were analyzed.

### Estimation framework

Health outcomes were assessed through four established indicators: age-standardized mortality rate (ASMR), DALYs, years of life lost (YLL), and years lived with disability (YLD) (29). Time-series forecasting was performed using Auto Regressive Integrated Moving Average (ARIMA) and Exponential Smoothing (ES) models in R. ARIMA model combines three core elements—Autoregressive (AR), Integrated (I), and Moving Average (MA)-to effectively capture linear trends and seasonal patterns in data. The ES model is based on an exponential weighted average, assuming that future trends are similar to historical patterns. The ES model employs an exponentially weighted average, operating on the premise that future trends will resemble past patterns (30). Both approaches projected ASMRs and DALYs for smoking-related MDR-TB through 2050 based on historical trends (31).

### Statistical analysis

All analyses were conducted in R (4.4.1), with statistical significance set at *α* = 0.05. Temporal trends in age-standardized mortality rate(ASMR) and age-standardized disability-adjusted life-year rate (ASDR) (1990–2021) were assessed using estimated annual percentage change (EAPC) modeling, where the natural log-transformed ASR follows the linear model ln(*y*) = *α* + *βx* + *ε*. The EAPC (calculated as 100 × (e^*β* − 1)) with its 95% CI determined trend significance: increasing when both EAPC and CI lower bound >0, decreasing when upper bound <0. For GBD 2021 estimates, we evaluated statistical differences through 95% uncertainty interval (UI) overlap-non-overlapping UIs indicated significant differences (*p* < 0.05).

## Results

### Global burden of MDR-TB attributable to smoking from 1990 to 2021 by ASR and EAPC

**Figure 1:**
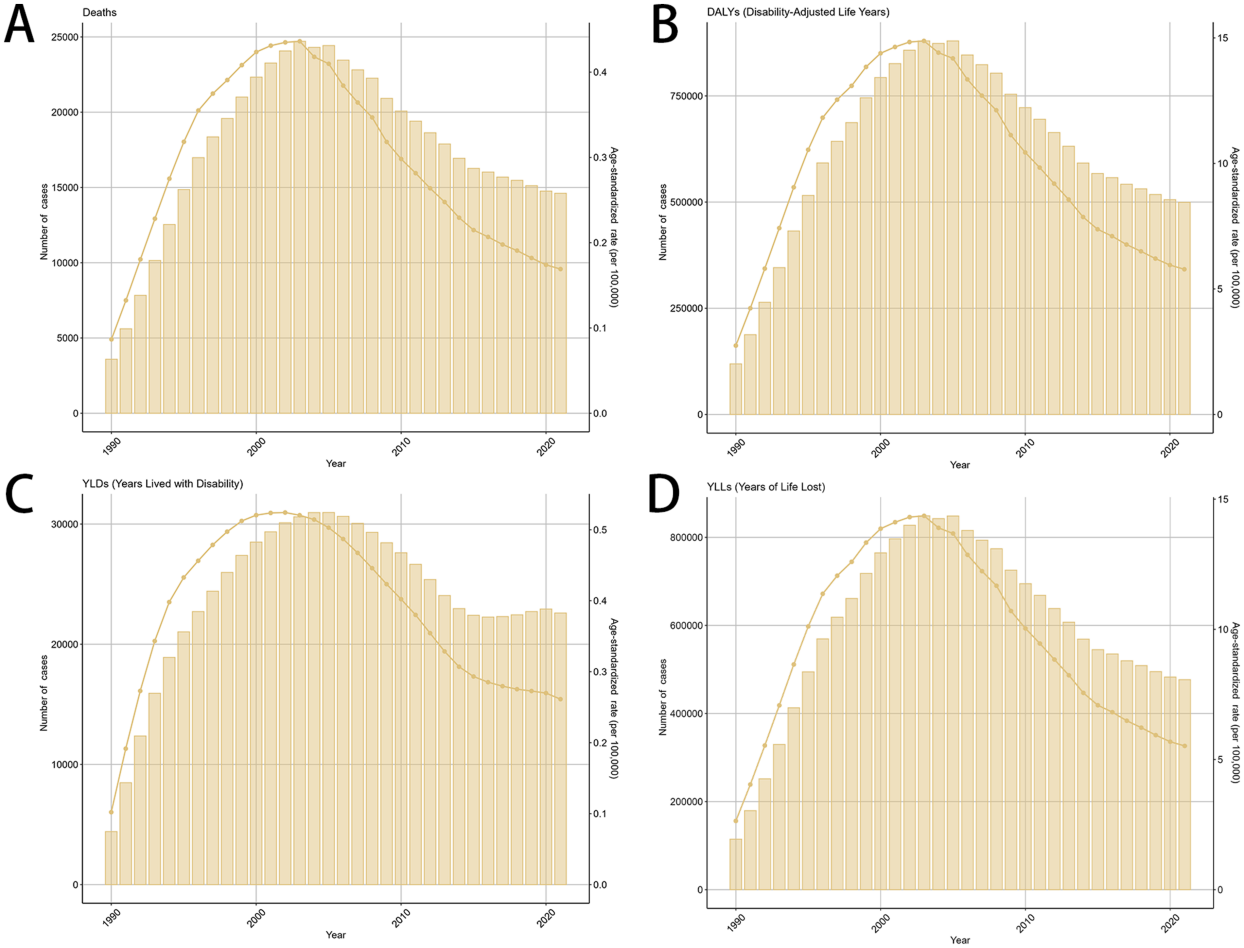
The global trends in the burden of smoking-related MDR-TB from 1990 to 2021. **(A)** Number of death case, **(B)** DALYs, **(C)** YLDs, **(D)** YLLs. DALY, disability-adjusted life years. YLDs, years lived with disability; YLLs, years of life lost.

Globally, the death number of MDR-TB cases attributable to Smoking was 3,589 (1,230–9,173) in 1990 and 14,610 (5,350–30,600) in 2021, with an ASR of incidence of 0.09 (0.03–0.22) in 1990 and 0.17 (0.06–0.35) in 2021. This rate decreased by an average of −0.06% (−1.13–1.01%) per year from 1990 to 2021 (EAPC) (Table 1). The number of new cases of MDR-TB attributable to Smoking has been decreasing globally from 1990 to 2021 (Figures 1A–D).

**Table 1:** The death burden of MDR-TB attributable to smoking in 1990 and 2021 and the temporal trends from 1990 to 2021.

Characteristics		1990		2021		EAPC_CI
		ASR (95%UI)	No. (95%UI)	ASR (95%UI)	No. (95%UI)	
Global		0.09 (0.03–0.22)	3,589 (1,230–9,173)	0.17 (0.06–0.35)	14,610 (5,350–30,600)	−0.06 (−1.13 to 1.01)
Sex						
	Female	0.01 (0–0.04)	307 (106–771)	0.03 (0.01–0.07)	1,411 (451–3,197)	0.11 (−1.26 to 1.51)
	Male	0.17 (0.06–0.44)	3,281 (1,120–8,419)	0.32 (0.12–0.67)	13,200 (4,915–27,455)	−0.77 (−2.26 to 0.75)
Age						
	30–34 years	0.04 (0.02–0.11)	164 (59–413)	0.12 (0.05–0.24)	703 (280–1,422)	0.33 (−1.54 to 2.24)
	35–39 years	0.06 (0.02–0.16)	220 (76–567)	0.16 (0.06–0.35)	906 (351–1981)	0.37 (−1.29 to 2.06)
	40–44 years	0.09 (0.03–0.22)	247 (84–638)	0.23 (0.09–0.46)	1,165 (434–2,326)	0.19 (−1.5 to 1.91)
	45–49 years	0.12 (0.04–0.31)	281 (98–710)	0.3 (0.12–0.61)	1,423 (573–2,893)	−0.19 (−2.05 to 1.7)
	50–54 years	0.17 (0.06–0.45)	371 (130–955)	0.37 (0.14–0.76)	1,628 (626–3,391)	−0.35 (−2.03 to 1.37)
	55–59 years	0.23 (0.08–0.6)	435 (151–1,109)	0.46 (0.18–1.01)	1,839 (696–3,994)	−0.35 (−1.78 to 1.1)
	60–64 years	0.3 (0.1–0.74)	478 (157–1,189)	0.57 (0.2–1.2)	1,812 (650–3,842)	−0.82 (−2.26 to 0.65)
	65–69 years	0.4 (0.13–1.04)	491 (157–1,282)	0.58 (0.2–1.27)	1,605 (556–3,497)	−1.51 (−2.86 to 0.14)
	70–74 years	0.49 (0.15–1.26)	413 (131–1,069)	0.65 (0.22–1.43)	1,336 (452–2,949)	−1.56 (−2.79 to 0.3)
	75–79 years	0.45 (0.15–1.18)	278 (91–723)	0.74 (0.24–1.65)	975 (320–2,172)	−1.4 (−2.67 to 0.11)
	80–84 years	0.4 (0.14–0.99)	140 (48–349)	0.79 (0.25–1.87)	692 (215–1,634)	−0.61 (−2 to 0.8)
	85–89 years	0.37 (0.13–0.92)	56 (20–139)	0.81 (0.26–1.83)	368 (118–835)	−0.46 (−1.83 to 0.93)
	90–94 years	0.27 (0.1–0.6)	11 (4–26)	0.74 (0.23–1.65)	132 (42–295)	−0.08 (−1.47 to 1.33)
	95+ years	0.23 (0.09–0.5)	2 (1–5)	0.5 (0.17–1.03)	27 (9–56)	−0.96 (−2.37 to 0.48)
SDI						
	Low	0.07 (0.02–0.17)	169 (53–416)	0.44 (0.15–0.96)	2,420 (869–5,227)	2.83 (0.85–4.86)
	Low-middle	0.04 (0.01–0.12)	268 (72–813)	0.53 (0.15–1.27)	7,907 (2,265–18,815)	4.3 (1.74–6.93)
	Middle	0.17 (0.05–0.49)	1,896 (502–5,286)	0.1 (0.04–0.21)	2,725 (1014–5,674)	−4.61 (−5.75 to 3.44)
	High-middle	0.1 (0.03–0.29)	1,045 (271–3,020)	0.08 (0.04–0.14)	1,469 (799–2,575)	−3.59 (−5.44 to 1.7)
	High	0.02 (0.01–0.04)	210 (92–439)	0 (0–0.01)	85 (32–190)	−7.46 (−8.48 to 6.44)
GBD regions						
	Oceania	0.01 (0–0.04)	0 (0–1)	0.32 (0.07–0.81)	30 (6–77)	10.51 (9.19–11.86)
	Southeast Asia	0.09 (0.02–0.26)	233 (61–674)	0.17 (0.06–0.36)	1,123 (389–2,352)	−1.06 (−2.45 to 0.35)
	East Asia	0.29 (0.07–0.79)	2,581 (608–7,113)	0.05 (0.01–0.13)	1,071 (254–2,905)	−7.85 (−8.67 to 7.03)
	Central Europe	0.02 (0.01–0.04)	26 (8–58)	0.01 (0–0.02)	16 (5–35)	−4.23 (−5.25 to 3.19)
	Central Asia	0.01 (0–0.04)	6 (1–19)	0.27 (0.14–0.44)	264 (141–430)	5.03 (2.19–7.94)
	Eastern Europe	0.05 (0.02–0.14)	139 (43–387)	0.33 (0.18–0.5)	973 (534–1,445)	2.14 (−0.07 to 4.4)
	Australasia	0 (0–0)	0 (0–1)	0 (0–0)	0 (0–1)	0.06 (0.02–0.13)
	High-income Asia Pacific	0.03 (0.01–0.08)	52 (13–159)	0 (0–0.01)	18 (3–57)	−8.83 (−10.09 to 7.56)
	Southern Latin America	0.01 (0–0.03)	4 (1–13)	0.01 (0–0.02)	5 (1–15)	−3.48 (−4.43 to 2.52)
	Western Europe	0.01 (0–0.01)	35 (13–70)	0 (0–0)	17 (6–35)	−4.58 (−5.38 to 3.77)
	High-income North America	0.01 (0–0.03)	40 (15–87)	0 (0–0)	5 (1–13)	−8.4 (−9.38 to 7.42)
	Caribbean	0.01 (0–0.03)	3 (1–9)	0 (0–0.01)	2 (1–7)	−5.14 (−5.85 to 4.43)
	Andean Latin America	0.07 (0.01–0.19)	15 (3–43)	0.08 (0.03–0.18)	48 (16–108)	−2.27 (−3.35 to 1.19)
	Tropical Latin America	0 (0–0.01)	2 (0–10)	0.02 (0–0.07)	56 (11–173)	3.41 (1.36–5.5)
	Central Latin America	0 (0–0.01)	4 (1–11)	0.01 (0–0.03)	34 (11–79)	−0.58 (−2.12 to 0.99)
	South Asia					
	North Africa and Middle East	0.01 (0–0.03)	21 (6–55)	0.03 (0.01–0.07)	151 (51–336)	0.93 (−0.69 to 2.58)
	Central Sub-Saharan Africa	0.09 (0.02–0.29)	23 (5–79)	0.33 (0.08–1)	235 (56–730)	1.63 (0.38–2.9)
	Western Sub-Saharan Africa	0.04 (0.01–0.1)	41 (13–96)	0.13 (0.05–0.29)	303 (104–661)	0.53 (−0.97 to 2.06)
	Eastern Sub-Saharan Africa	0.03 (0.01–0.09)	26 (8–70)	0.45 (0.16–0.94)	901 (315–1879)	4.4 (2.32–6.52)
	Southern Sub-Saharan Africa	0.16 (0.03–0.52)	51 (8–169)	0.64 (0.22–1.5)	434 (147–1,009)	3.37 (2.03–4.73)

ASDR, age-standardized death rate; EAPC, estimated annual percentage change; MDR-TB, multidrug-resistant tuberculosis; SDI, sociodemographic index; and UI, uncertainty interval.

Smoking-related MDR-TB led to >100,000 DALYs in 1990 and to 499,422 DALYs in 2021 globally. Compared with 1990, the burden of MDR-TB attributable to smoking has increased in terms of absolute numbers in 2021. The ASR per 100,000 people of MDR-TB attributable to smoking increased from 2.74 (95%UI, 0.94–6.92) in 1990 to 5.78(95%UI, 2.26–11.93) in 2021. There were increased YLDs [ASR, 0.26 (95%CI, 0.13–0.49)] and age-standardized YLL rate [ASR, 5.52 (95% CI, 2.11 to 11.33)] in 2021. Correspondingly, from 1990 to 2021, there were increase of YLDs [EAPC, 0.14 (95% CI, −0.82 to 1.12)], and YLLs [EAPC, 0.06 (95% CI, −1.05 to 1.19)] in the global, respectively, (Table 2, Supplementary Tables S1, S2).

**Table 2:** The DALY burden of MDR-TB attributable to smoking in 1990 and 2019 and the temporal trends from 1990 to 2021.

Characteristics		1990		2021		EAPC_CI
		ASR (95%UI)	No. (95%UI)	ASR (95%UI)	No. (95%UI)	
Global		2.74 (0.94–6.92)	119,061 (40,947–300,673)	5.78 (2.26–11.93)	499,422 (194,864–1,030,642)	0.07 (−1.04 to 1.19)
Sex						
	Female	0.41 (0.15–1.02)	9,112 (3,246–22,570)	0.98 (0.34–2.16)	43,795 (15,191–96,702)	0.39 (−1.07 to 1.87)
	Male	5.22 (1.8–13.23)	109,949 (37,878–278,265)	10.77 (4.25–22.13)	455,627 (180,270–934,568)	−0.52 (−2.07 to 1.07)
Age						
	30–34 years	2.57 (0.94–6.37)	9,891 (3626–24,542)	7.07 (2.96–14.08)	42,728 (17903–85,111)	0.35 (−1.51 to 2.23)
	35–39 years	3.44 (1.2–8.81)	12,105 (4219–31,024)	8.92 (3.49–19.36)	50,020 (19587–108,592)	0.37 (−1.28 to 2.05)
	40–44 years	4.27 (1.49–10.83)	12,229 (4267–31,035)	11.6 (4.5–23.11)	58,006 (22495–115,624)	0.19 (−1.49 to 1.9)
	45–49 years	5.38 (1.91–13.58)	12,484 (4,429–31,531)	13.47 (5.57–27.34)	63,785 (26,351–129,478)	−0.18 (−2.03 to 1.7)
	50–54 years	6.86 (2.42–17.46)	14,585 (5150–37,120)	14.55 (5.8–29.99)	64,741 (25787–133,433)	−0.34 (−2.02 to 1.37)
	55–59 years	8.14 (2.85–20.68)	15,070 (5,285–38,294)	16.26 (6.34–35.08)	64,332 (25,108–138,805)	−0.35 (−1.77 to 1.1)
	60–64 years	8.9 (2.96–21.94)	14,301 (4,753–35,244)	17.07 (6.24–35.86)	54,642 (19,969–114,768)	−0.81 (−2.25 to 0.65)
	65–69 years	10.1 (3.25–25.74)	12,488 (4,017–31,812)	15.01 (5.57–32.11)	41,395 (15,360–88,568)	−1.5 (−2.84 to 0.14)
	70–74 years	10.3 (3.26–26.09)	8,716 (2,760–22,085)	13.95 (4.97–30.02)	28,723 (10,225–61,801)	−1.55 (−2.78 to 0.31)
	75–79 years	7.58 (2.53–19.62)	4,664 (1,560–12,075)	12.6 (4.26–28.01)	16,620 (5,622–36,938)	−1.39 (−2.66 to 0.1)
	80–84 years	5.17 (1.8–12.83)	1,829 (637–4,538)	10.45 (3.39–24.57)	9,152 (2,965–21,522)	−0.61 (−1.99 to 0.8)
	85–89 years	3.81 (1.38–9.31)	576 (208–1,407)	8.43 (2.76–18.77)	3,853 (1260–8,583)	−0.43 (−1.8 to 0.96)
	90–94 years	2.39 (0.96–5.38)	103 (41–231)	6.67 (2.16–14.59)	1,194 (386–2,610)	−0.06 (−1.45 to 1.36)
	95+ years	1.96 (0.82–4.22)	20 (8–43)	4.25 (1.5–8.65)	231 (82–471)	−0.9 (−2.32 to 0.55)
SDI						
	Low	2.14 (0.69–5.2)	5,900 (1,901–14,366)	13.23 (4.83–28.28)	84,353 (31,919–179,781)	2.71 (0.71–4.76)
	Low-middle	1.26 (0.34–3.78)	9,274 (2,507–27,787)	16.12 (4.67–37.62)	263,593 (76,433–614,318)	4.27 (1.72–6.89)
	Middle	5.2 (1.41–14.28)	62,615 (17,068–171,342)	3.25 (1.27–6.71)	91,793 (35,989–189,995)	−4.3 (−5.45 to 3.12)
	High-middle	3.32 (0.91–9.33)	34,768 (9,550–97,875)	3.2 (1.79–5.27)	57,021 (32,009–94,925)	−2.97 (−4.95 to 0.95)
	High	0.62 (0.28–1.28)	6,471 (2,933–13,284)	0.16 (0.07–0.34)	2,511 (1023–5,413)	−7.1 (−8.07 to 6.12)
GBD regions						
	Oceania	0.33 (0.66–1.24)	13 (2–49)	11.39 (2.45–29.47)	1,217 (263–3,174)	10.72 (9.5–11.96)
	Southeast Asia	2.5 (0.66–7.35)	7,347 (1,968–22,384)	4.93 (1.79–10.12)	35,490 (12,706–72,445)	−1.06 (−2.41 to 0.31)
	East Asia	8.5 (2–23.33)	84,331 (19,766–231,721)	1.64 (0.41–4.4)	35,179 (8,764–94,599)	−7.68 (−8.47 to 6.87)
	Central Europe	0.68 (0.22–1.54)	982 (315–2,202)	0.38 (0.13–0.82)	617 (210–1,314)	−4.25 (−5.26 to 3.23)
	Central Asia	0.46 (0.12–1.44)	250 (64–778)	11.27 (6.2–18.28)	11,345 (6252–18,450)	4.98 (2.11–7.93)
	Eastern Europe	2.06 (0.66–5.73)	5,465 (1,744–15,247)	14.33 (8.08–20.88)	40,006 (22,561–58,433)	2.3 (0.06–4.58)
	Australasia	0 (0–0)	0 (0–1)	0.03 (0.01–0.07)	12 (3–30)	−1.02 (−1.57 to 0.47)
	High-income Asia Pacific	0.74 (0.18–2.36)	1,512 (370–4,826)	0.09 (0.02–0.29)	343 (70–1,090)	−9.62 (−10.8 to 8.43)
	Southern Latin America	0.34 (0.08–1.06)	159 (38–496)	0.23 (0.04–0.69)	178 (35–540)	−3.49 (−4.4 to 2.56)
	Western Europe	0.18 (0.07–0.37)	944 (358–1,896)	0.06 (0.02–0.12)	412 (171–825)	−4.61 (−5.27 to 3.95)
	High-income North America	0.39 (0.15–0.85)	1,258 (496–2,736)	0.03 (0.01–0.07)	138 (38–373)	−8.37 (−9.34 to 7.39)
	Caribbean	0.4 (0.11–1.17)	115 (32–335)	0.16 (0.04–0.47)	84 (19–244)	−5.2 (−5.9 to 4.49)
	Andean Latin America	2.2 (0.5–6.27)	546 (125–1,545)	2.64 (0.93–5.78)	1,694 (595–3,689)	−2.31 (−3.36 to 1.24)
	Tropical Latin America	0.08 (0.01–0.32)	87 (11–366)	0.74 (0.14–2.31)	1,969 (383–6,146)	3.23 (1.19–5.32)
	Central Latin America	0.15 (0.04–0.39)	151 (45–390)	0.45 (0.14–1.01)	1,190 (380–2,658)	−0.44 (−1.93 to 1.07)
	South Asia	1.4 (0.27–4.58)	9,867 (1,889–32,597)	17.73 (4.31–43.23)	293,950 (70,950–715,811)	5 (3.09–6.94)
	North Africa and Middle East	0.34 (0.11–0.9)	687 (212–1,784)	0.95 (0.33–2.13)	5,431 (1,842–12,656)	1.23 (−0.36 to 2.86)
	Central Sub-Saharan Africa	2.94 (0.6–9.77)	878 (178–2,939)	11.4 (2.76–35.22)	9,276 (2,243–29,023)	1.79 (0.65–2.95)
	Western Sub-Saharan Africa	1.38 (0.45–3.23)	1,501 (486–3,548)	4.18 (1.5–8.97)	11,180 (4,002–23,939)	0.49 (−0.9–1.9)
	Eastern Sub-Saharan Africa	0.97 (0.31–2.64)	902 (293–2,448)	14.27 (5.14–29.6)	33,447 (12,061–69,506)	4.58 (2.61–6.59)
	Southern Sub-Saharan Africa	5.83 (0.95–19.5)	2,058 (334–6,869)	22.32 (7.61–52.01)	16,264 (5,545–38,003)	3.2 (1.89–4.52)

ASDAR, age-standardized DALYs rates; DALYs, disability-adjusted life-year; EAPC, estimated annual percentage change; MDR-TB, multidrug-resistant tuberculosis; UI, uncertainty interval.

### Global burden of MDR-TB attributable to smoking by sex

**Figure 2:**
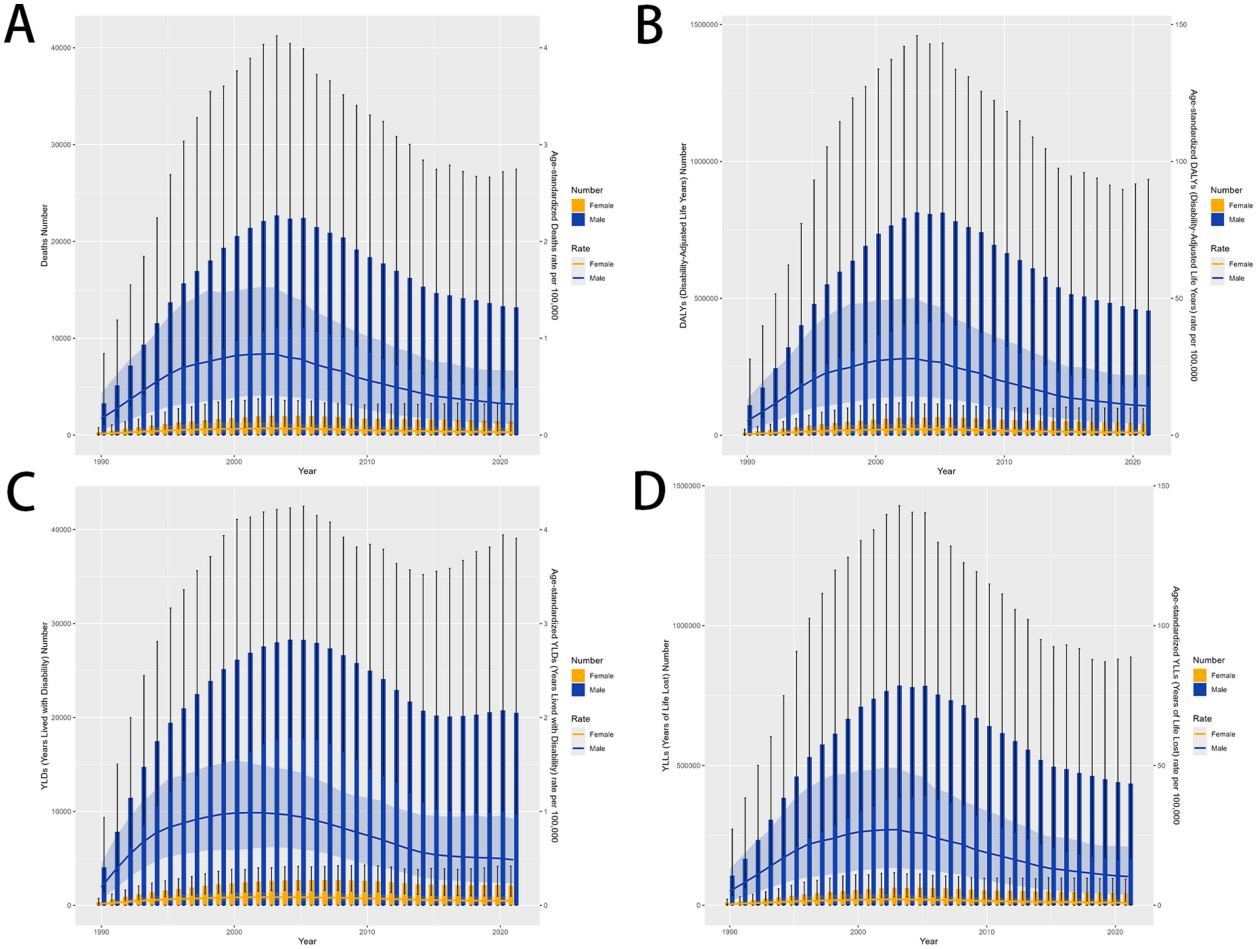
MDR-TB due to smoking-related GBD of deaths, YLDs, YLLs and DALYs between for sex differences between 1990 and 2021. The trends in males and females of number of case **(A)** Deaths **(B)** DALYs **(C)** YLDs **(D)** YLLs. DALYs, disability-adjusted life years. YLDs, Years lived with Disability. YLLs, Years of life lost.

There was a significant sex disparity in the smoking-related MDR-TB burden, in which the smoking-related MDR-TB burden was approximately ten times higher in men than that in women. In absolute terms, this burden led to 13,200 deaths in men, significantly more than in women (≈1,411 deaths) (Figure 2A). Similarly, DALYs, YLDs, and YLLs were higher for men [455,627 (95%UI, 180270–934,568)], [20,485 (95%UI, 9772–39,066)], and [435,143 (95%UI, 169066–887,423)] than for women [43,795 (95% UI, 15191–96,702)], [2,116 (95% UI, 983–4,186)], and [41,679 (95% UI, 13857–93,133)] (Figures 2B–D). The ASMRs per 100,000 people of MDR-TB attributable to smoking in women and men were 0.03 (95% UI, 0.01–0.07) per 100,000 and 0.32 (95% UI, 0.12–0.67) per 100,000, respectively [EAPC, 0.11 (95% CI, −1.26 to 1.51)] and −0.77 [95% CI, −2.26 to 0.75]. ASDRs per 100,000 people were 0.98 (95% UI, 0.34–2.16) per 100,000 and 10.77 (95% UI, 4.25–22.13) per 100,000 in women and men, respectively [EAPC, 0.39 (95% CI, −1.07 to 1.87) and −0.52 (95% CI, −2.07 to 1.07)]. YLDs per 100,000 people were 0.02 (95% UI, 0.01–0.03) per 100,000 and 0.05 (95% UI, 0.02–0.09) per 100,000 in women and men, respectively [EAPC, 0.63 (95% CI, −0.75 to 2.02) and −0.48 (95% CI, −1.83 to 0.89)]. YLLs per 100,000 people were 0.4 (95% UI, 0.14–1) per 100,000 and 0.94 (95% UI, 0.31–2.08) per 100,000 in women and men, respectively [EAPC, 0.38 (95% CI, −1.09 to 1.87) and −0.52 (95% CI, −2.08 to 1.08)]. Overall, from 1990 to 2021, the Global Burden of Disease attributable to smoking trended downward in both sexes. However, women have experienced a more pronounced decrease. Detailed results are provided in Tables 1, 2.

### Smoking-related MDR-TB burden in age groups

**Figure 3:**
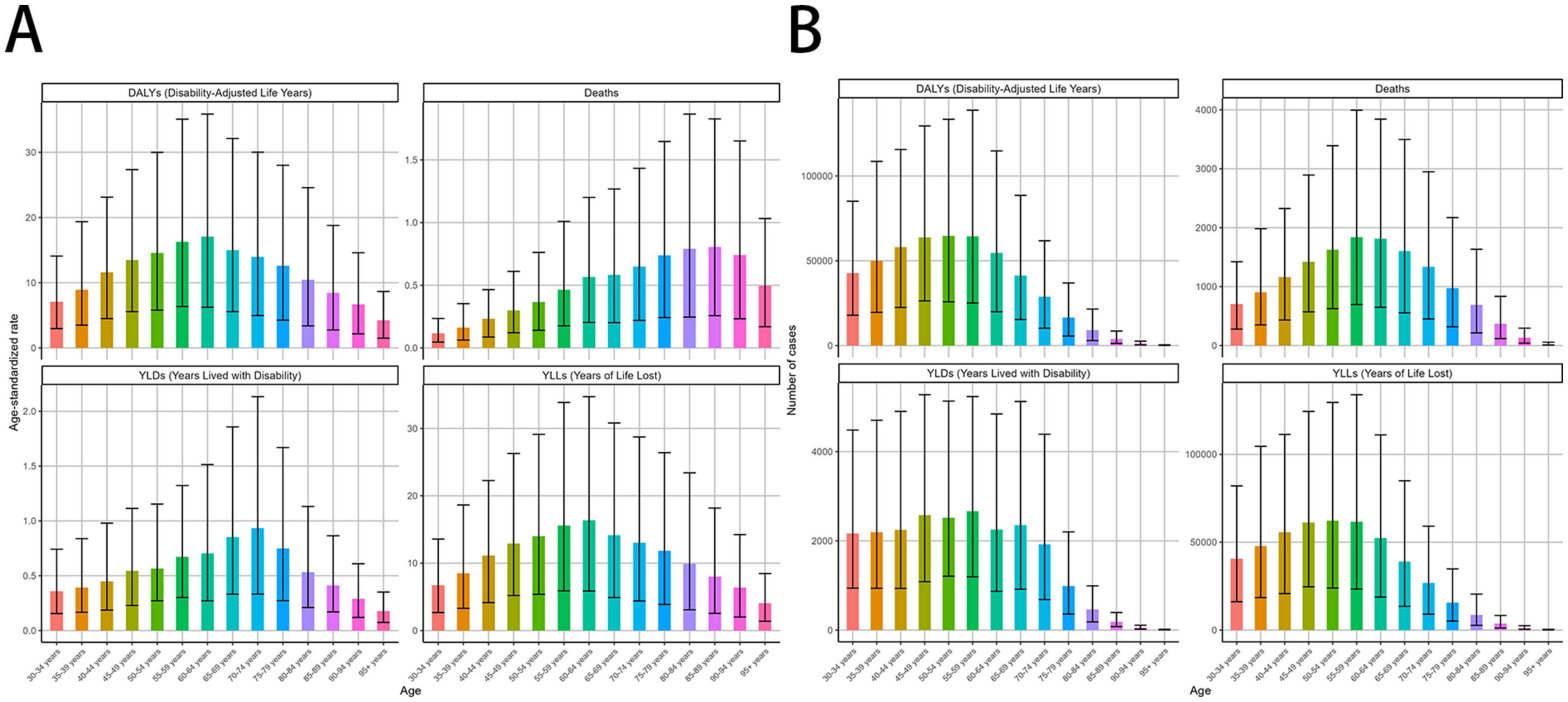
MDR-TB due to smoking-related GBD of deaths, YLDs, YLLs and DALYs between for different ages between 1990 and 2021. Age-specific distributions of disease burden metrics in 2021. Age-standardized rate. **(A)** DALYs, Deaths, YLDs, YLLs. Number of case **(B)** DALYs, Deaths, YLDs, YLLs. DALYs, disability-adjusted life years. YLDs, Years lived with Disability. YLLs, Years of life lost.

Smoking-attributable death and DALYs for MDR-TB followed a consistent global trend. In 2021, death for the high populations aged 55–59, 60–64 years were 1839 (95% CI: 696–3,994), 1812 (95% CI: 650–3,842) per 100,000, respectively, DALYs for the high populations aged 50–54, 55–59 years were 64,741 (95% CI, 25787–133,433), 64,332 (95% CI, 25,108–138,805) per 100,000, respectively (Figures 3A–D), and a global decline in smoking-attributable death and DALYs across most age groups was observed (Tables 2).

According to the analysis of changing trends in disease burden stratified by age, from 1990 to 2021, DALYs, and YLLs among different age subgroups exhibited a consistent distribution pattern, with a unimodal distribution of initially increasing and then decreasing. The mortality showed high levels in the age groups of 30–34 years, 35-39 years, 40–44 years, with a peak in the 35–39 years age group of 0.37 (95% UI −1.29 to 2.06) per 100,000 population, Similar patterns were observed for DALYs, and YLLs (Tables 1, 2).

### The burden of MDR-TB attributable to smoking by SDI super-region

**Figure 4:**
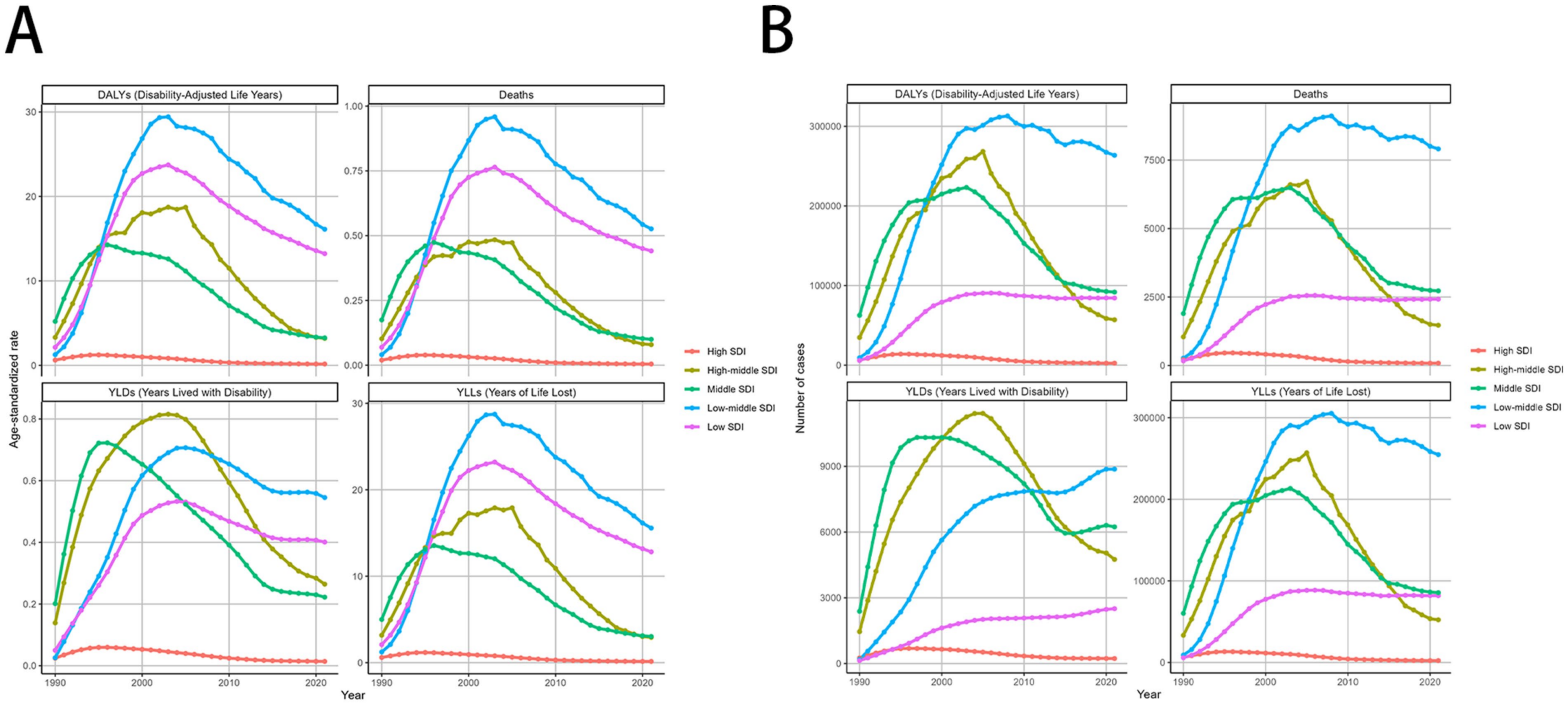
MDR-TB due to smoking-related GBD of deaths, YLDs, YLLs and DALYs between for different SDI regions between 1990 and 2021. Line graphs of 1990-2021 trends in age- standardized **(A)** DALYs, deaths, YLDs, and YLLs. Number of case **(B)** DALYs, Deaths, YLDs, YLLs. DALYs, disability-adjusted life years. YLDs, Years lived with Disability. YLLs, Years of life lost.

The burden of MDR-TB attributable to smoking varied with SDI-defined super-regions. In 2021, this burden was greatest in countries with Low-middle SDI, with an ASMR and ASDR per 100,000 people of 0.53 (95% UI, 0.15–1.27) and 16.12 (95% UI, 4.67–37.62), respectively. Conversely, the least burden was seen in high-SDI countries, where ASMR and ASDR per 100,000 people were 0 (95% UI, 0–0.01) and 0.16 (95% UI, 0.07–0.34), respectively. Over time, the 3 SDI super-regions had downward trending MDR-TB burdens attributable to smoking. The most significant decrease was seen in high-SDI countries, with EAPCs of −7.46 (95% CI, −8.48 to −6.44) and −7.1 (95% CI, −8.07 to −6.12), respectively. In contrast, high-middle SDI countries experienced the least change over time, with EAPCs of −3.59 (95% CI, −5.44 to −1.7) and −2.97 (95% CI, −4.95 to −0.95), respectively. Detailed results are provided in Tables 1, 2; Figures 4A–D.

At the regional level, in 2021, Australasia had the lowest ASMR [0 (95% UI: 0–1)] and ASDR [12 (95% UI: 3–30)], while Central Europe had the highest ASMR [4.13 (95% UI: 1.71–6.20)] and ASDR [92.49 (95% UI: 38.15–138.76)] (Figure 4A). In 2021, East Asia had the highest number of MDR-TB deaths [1,123 (95% UI: 389–2,352)], However, DALYs [293,950 (95% UI: 70,950–715,811)] of South Asia attributable to smoking had the highest number in 2021, followed by Eastern Europe and Southeast Asia (Figure 4B). High-income Pacific showed the largest decrease in the ASMR of MDR-TB attributable to smoking, with an EAPC of −8.83 (95% CI: −10.09 to −7.56) in ASMR and an EAPC of −9.62 (95% CI: −10.8 to −8.43) in ASDR. While, Oceania showed the largest increase with an EAPC of 10.51 (95% CI: 9.19–11.86) in ASMR and an EAPC of 10.72 (95% CI: 9.5–11.96) in ASDR (Tables 1, 2).

### Burden of MDR-TB attributable to smoking by country

**Figure 5:**
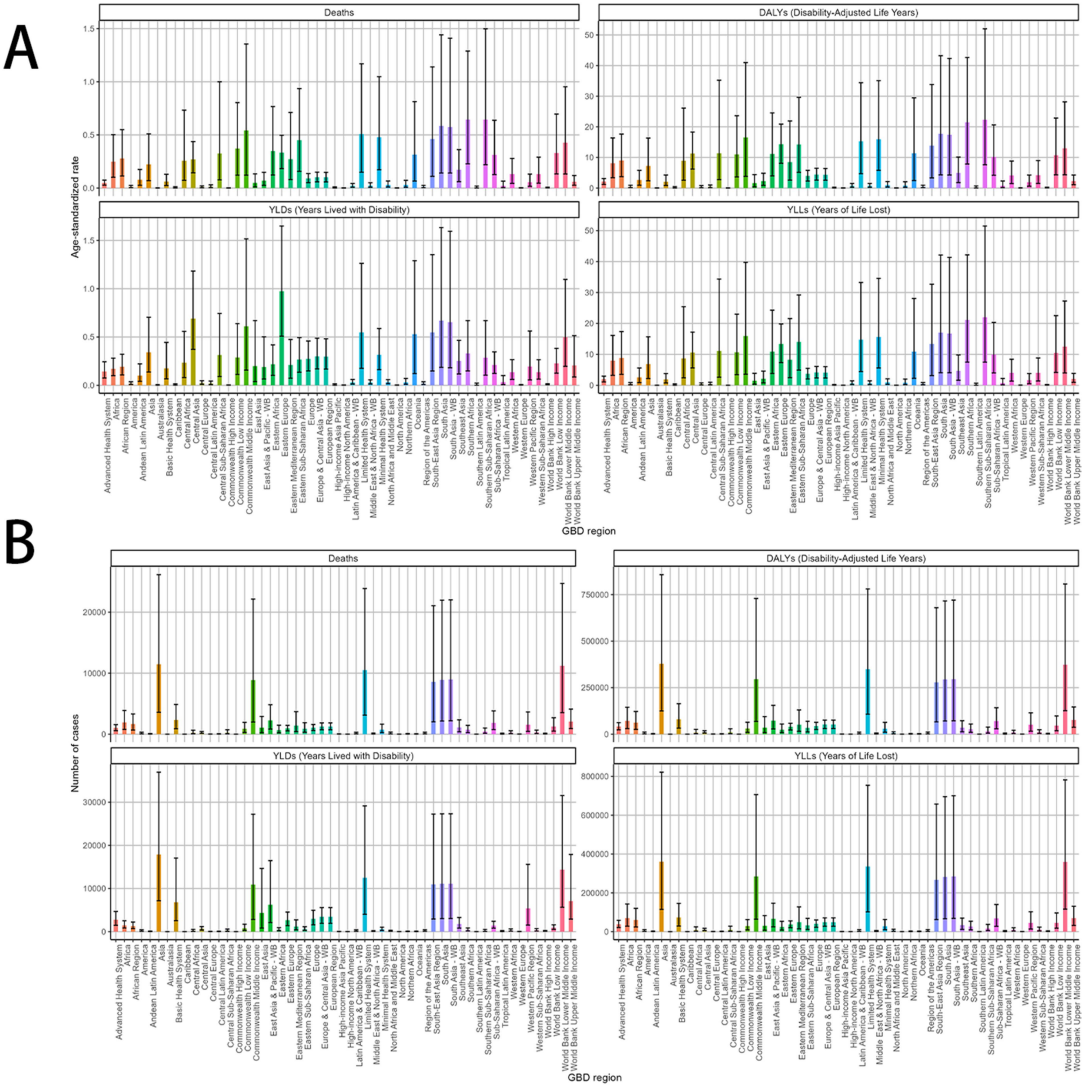
MDR-TB due to smoking-related GBD of deaths, YLDs, YLLs and DALYs between for different GBD country. A 2021 multivariate data portrayal via a polychromatic bar chart. **(A)** DALYs, deaths, YLDs, YLLs. Number of case **(B)** DALYs, deaths, YLDs, YLLs. DALYs, disability-adjusted life years; YLDs, years lived with disability; YLLs, years of life lost.

Specifically, in both 1990 and 2021, the burden of MDR-TB attributable to smoking was extremely variable among countries. In 2021, ASRs of smoking-related MDR-TB burden varied as much as 50-fold between countries, with ASDRs and ASDARs per 100,000 people 4.45 (95%UI, 0.99–13.52) and 156.12 (95%UI, 34.26–471.88) in the Lesotho, respectively. In 2021, India (South Asia), Russia Federation (Eastern Europe), Pakistan (South Asia), and China (Eastern Asia) led in the number of DALYs across 204 countries and territories. India topped the list with 239,707 cases, ahead of Russia with 26,111 and China with 27,421 cases (Figure 5). Additionally, the top 3 countries by case count include Africa nations: Somalia, Zimbabwe, Mozambique, had the greatest absolute number of deaths and DALYs attributable to smoking-related MDR-TB because of their large populations.

**Figure 6:**
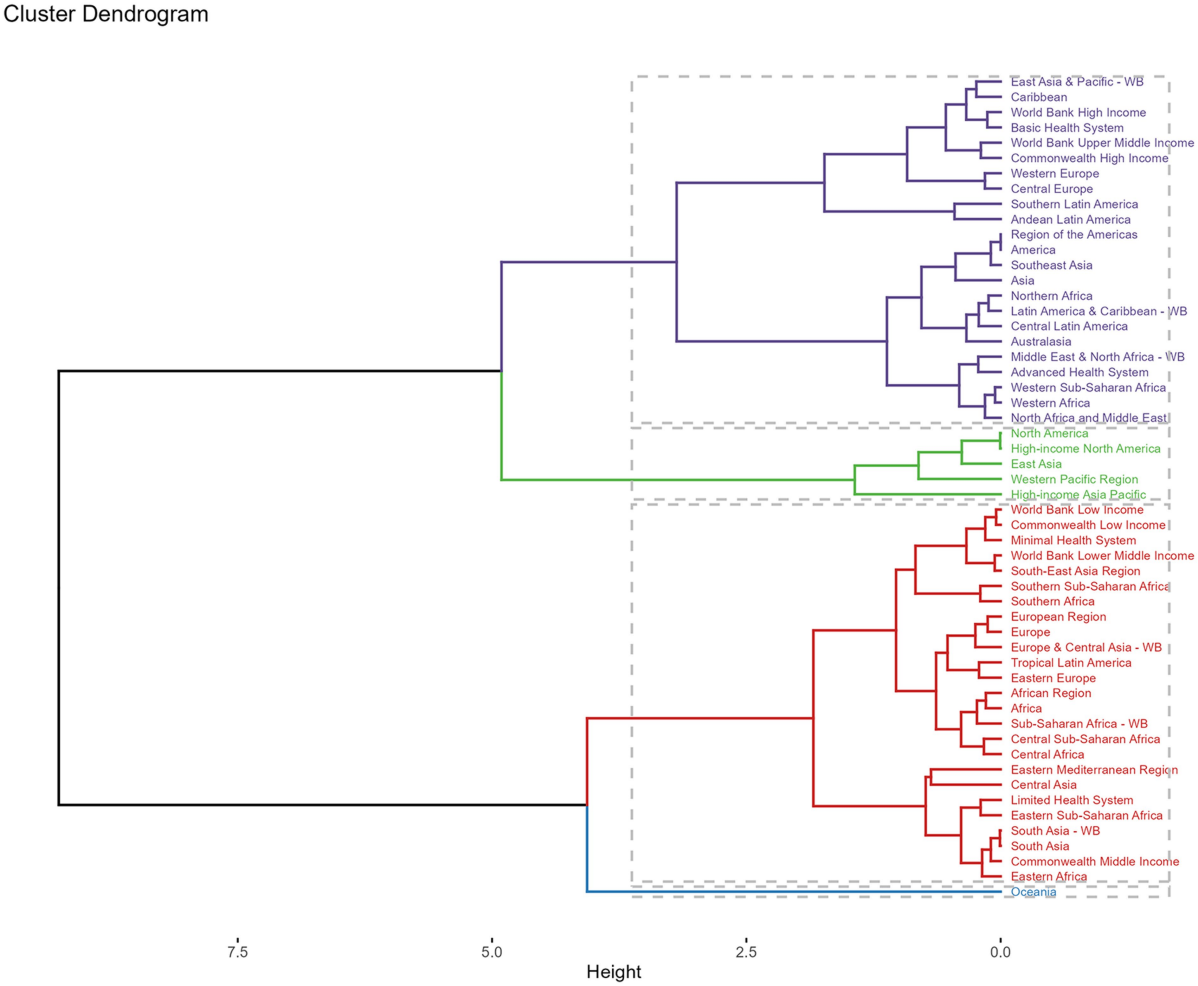
Results of cluster analysis based on GBD in 2021. Purple line: minor increase, green line: remained stable or minor decrease, red line: significant decrease, blue line: significant increase. DALYs, disability-adjusted-life-years.

Across GBD regions, the MDR-TB attributable to Smoking burden varied. To find regions with similar variation in disease burden, a hierarchical clustering analysis was conducted in this study. The results were shown in Figure 6. The significant deaths rate and DALYs rate increase occurred in Oceania, while the significant decrease was in World Bank Low Income, Commonwealth Low Income, Minimal Health System, World Bank Lower Middle Income, South-East Asia Region, Southern Sub-Saharan African, Southern Africa, European Region, Europe, Europe & Central Asia-WB, Tropical Latin America, Eastern Europe, African Region, African, Sub-Saharan Africa-WB, Central Sub-Saharan Africa, Central Africa, Eastern Mediterranean Region, Central Africa, Eastern Mediterranean Region, Central Asia, Limited Health System, Eastern Sub-Saharan Africa, South Asia-WB, South Asia, Commonwealth Middle Income, and Eastern Africa.

### Prediction of MDR-TB attributable to smoking from 2022 to 2050

**Figure 7:**
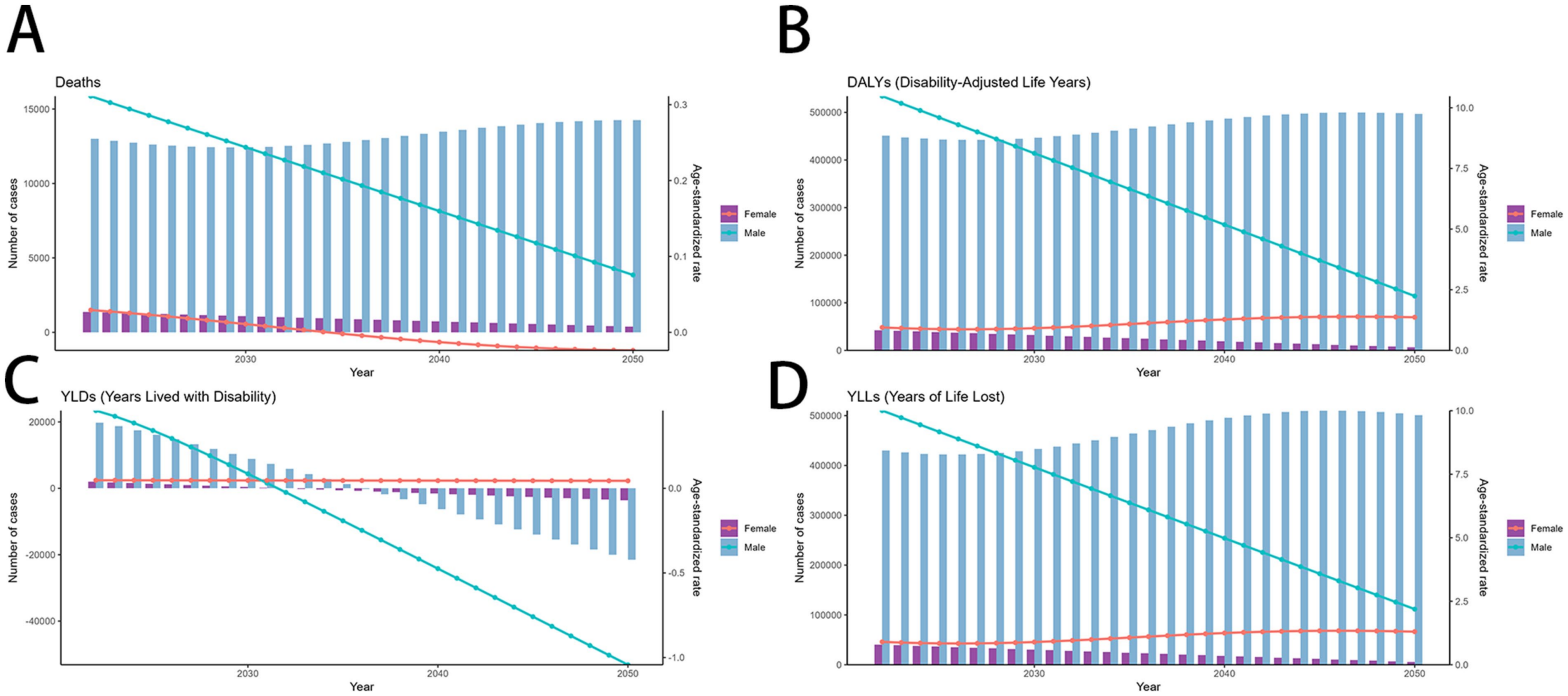
Predicted trends of smoking-related MDR-TB in an ARIMA model. **(A)** Deaths **(B)** DALYs, **(C)** YLDs, **(D)** YLLs. DALYs, disability-adjusted life years; YLDs, years lived with disability; YLLs, years of life lost.

**Figure 8:**
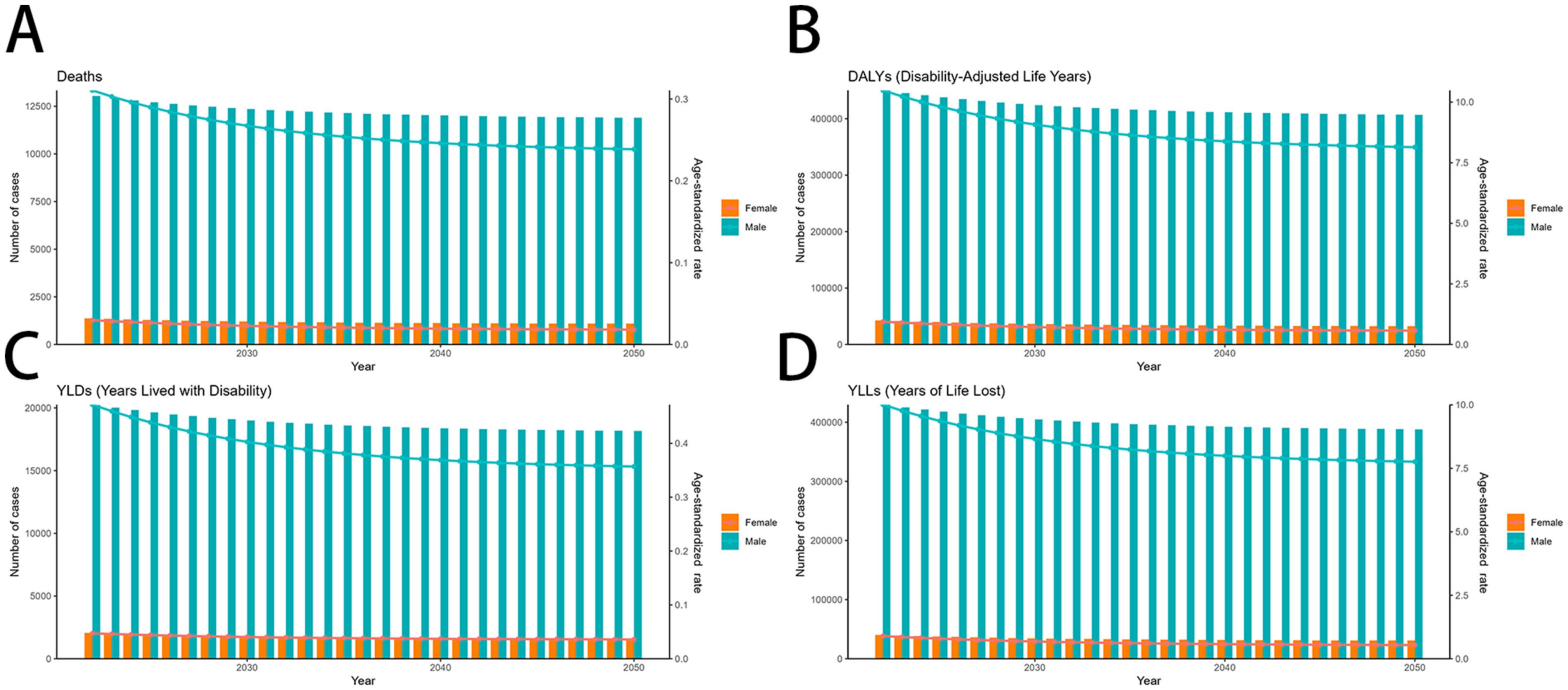
Predicted trends of smoking-related MDR-TB in an ES model **(A)** Deaths **(B)** DALYs, **(C)** YLDs, **(D)** YLLs. DALYs, disability-adjusted life years; YLDs, years lived with disability; YLLs, years of life lost.

Using the ARIMA model, global mortality due to smoking-related MDR-TB is projected to a downward trend from 2022 to 2050, while DALYs and YLLs continues to decline in male and increase slightly in female. Both sexes are expected to show stable in YLDs, with.

(Figures 7A–D). In contrast, In the ES model, MDR-TB among women is projected to stable marginally, this is a promising result. In contrast, MDR-TB cases in males showed a decline (Figures 8A–D). These projections underscore the need for continued surveillance and targeted interventions to mitigate the future burden of smoking-related MDR-TB.

## Discussion

This study aimed to elucidate the burden of multidrug-resistant tuberculosis (MDR-TB) attributable to smoking through an empirical analysis of Deaths, DALYs, YLDs, and YLLs between 1990 and 2021. The observed upward trajectory in both the absolute number of cases and the ASR indicates a growing public health challenge, necessitating targeted interventions to control the incidence of drug-resistant TB and mitigate its escalating impact.

While areas currently bearing the heaviest disease burden deserve priority attention, equal emphasis should be placed on regions where cases have risen most sharply in recent years. The sharpest rises in ASRs for mortality, DALYs, YLDs, and YLLs from 1990 through 2021 occurred in Oceania and Central Asia. Increasing funding for MDR-TB programs linked to tobacco control in these two areas could prove beneficial.

The analysis demonstrated a significant gender dichotomy in the tuberculosis burden attributable to tobacco use, with males exhibiting considerably higher rates than females. This disparity is potentially attributable to a combination of pathogenic mechanisms: Endogenous hormonal variations, specifically the putative role of female sex hormones in potentiating more robust immune responses to cytotoxic challenges. A heightened prevalence of modifiable risk factors (e.g., smoking) and a documented lower engagement with healthcare services among male populations. As can be seen from the results, the death burden of disease is relatively higher in all age groups 30–39. Individuals in young and middle adulthood usually have a fully developed and potent immune system. When *Mycobacterium tuberculosis* enters the body, the immune system responds with an intense assault to isolate and eradicate the pathogen. This reaction results in widespread inflammation and tissue death. Secondly, Owing to the aforementioned factors (e.g., fatigue and pressure), the immune function of young and middle-aged individuals becomes compromised, causing latent tuberculosis bacteria in the body to become active again and trigger ‘endogenous reactivation’—the main cause of adult tuberculosis. Studies have pointed out that smoking impairs the autophagy function of macrophages, and autophagy is essential for the removal of *Mycobacterium tuberculosis*. Autophagy defects caused by smoking may make it more difficult for macrophages to resist *Mycobacterium tuberculosis* infection, thereby increasing the risk of tuberculosis (32).

As of 2021, Lesotho exhibits the highest MDR-TB burden connected to smoking, while regions in Europe, the Americas, and Oceania report comparatively lower burdens. Concerning the distribution of MDR-TB cases by region, our results indicated that Lesotho, Uzbekistan, and Kyrgyzstan experienced the highest mortality rates. Lesotho, a small and landlocked nation encircled by South Africa, has a mortality rate of 650 per 100,000 population, with an estimated 11.8% of cases classified as MDR/RR-TB across both newly diagnosed and previously treated individuals. It is widely recognized that the high mortality rate results from the combined effects of tuberculosis, HIV, food scarcity, and poverty in Lesotho (33, 34). Uzbekistan ranks among the 30 countries worldwide burdened with high rates of MDR/RR-TB (35). Similarly, The Kyrgyz Republic is one of the high priority countries for MDR-TB, in 2021, 46% of the estimated 10.6 million people with TB were missed by health systems (known as “missed TB cases”) (36, 37). These areas have documented the highest TB incidence rates, which can largely be linked to economic challenges and undernutrition. Therefore, it is essential for these nations to emphasize the strategic allocation of healthcare resources, with particular attention to impoverished areas, efforts to combat hunger, and the development of social safety nets aimed specifically at assisting low-income families afflicted by TB.

Additionally, we implemented ARIMA and ES prediction models to estimate the impact of MDR-TB over the forthcoming 30 years. Projections indicate that by 2037, the mortality rate from MDR-TB linked to smoking among males is expected to decline, which implies effective control measures against smoking. Multiple factors contribute to the prevalence of MDR-TB, with five primary risk factors accounting for many TB cases globally: undernutrition, HIV infection, alcohol use disorder, diabetes, and smoking. Consequently, it is vital to acknowledge MDR-TB as a fundamental aspect in the realm of TB prevention and control. Attention should be directed not only toward minimizing TB incidence but also toward initiatives aimed at reducing the transition of drug-susceptible TB cases into drug-resistant variants.

To account for regional variations, governments should enact localized anti-smoking measures, such as hiking tobacco duties—a proven method to decrease demand (38, 39). As earnings increase, especially in developing nations, adapting tax systems is vital to sustain their efficacy. Whilst over 70 countries now have policies to ban smoking in public places, workplaces and public transport, Total bans seem to have a greater impact than partial bans (40). There can be concerns regarding how the laws are applied in practice, e.g., (41) as also seen in South Africa during the COVID-19 pandemic leading also to increase buying of cigarettes illicitly. In addition-return to previous practices as laws are relaxed (42–47). Potential ways forward include the use of NRT to help make quitting permanent-however, such policies are variably introduced across LMICs in view of cost/co-payment concerns (48).

Our findings provide valuable evidence to refine tobacco control policies and optimize healthcare delivery for vulnerable MDR-TB populations. It also emphasized how gender and age differences affect disease burden, underscoring the importance of targeted prevention strategies, thereby supporting the health-related Sustainable Development Goals. However, several methodological limitations should be considered when interpreting these results. Firstly, Reliance on self-reported smoking status from public repositories may result in prevalence underestimation, with particular under ascertainment among female populations. Secondly, the available datasets are limited to conventional tobacco formulations, omitting contemporary nicotine delivery systems such as electronic cigarettes, despite mounting evidence of their clinical consequences. Lastly, this investigation was constrained to evaluating active smoking-attributable MDR-TB burden due to unavailability of epidemiological data regarding passive smoke exposure, alternative nicotine consumption modalities, or electronic delivery systems.

## Conclusion

This research highlights significant worldwide decreases in the impact of MDR-TB linked to smoking, mainly driven by high-SDI areas implementing effective public health initiatives. Nevertheless, inequalities persist, especially in lower-SDI regions, among males, and within older age groups (ages 40–60), who encounter greater burdens due to increased smoking prevalence and restricted healthcare availability. To tackle these disparities, it is essential to develop targeted interventions tailored to specific regions to further mitigate the effects of smoking-related MDR-TB. Enhancing tobacco control, improving access to healthcare, and adopting gender-sensitive strategies are vital for attaining fair advancements in global tuberculosis health.

## Data Availability

The original contributions presented in the study are included in the article/Supplementary material, further inquiries can be directed to the corresponding author.
