# Supplementary material for: Global, regional, and national burdens of MDR-TB attributable to smoking from 1990 to 2021 with a prediction from 2022 to 2050
Source: Front Public Health. 2025 Sep 19;13:1634772. doi: 10.3389/fpubh.2025.1634772 (PMC12491277; doi:10.3389/fpubh.2025.1634772)
Supplement: Supplementary file 1 [file Supplementary_file_1.docx]

Table S1 The YLDs Burden of MDR-TB Attributable to Smoking in 1990 and 2021 and the Temporal Trends From 1990 to 2021

| Characteristics | | 1990 | | 2021 | |  |
| --- | --- | --- | --- | --- | --- | --- |
|  |  | ASR (95%UI) | No.(95%UI) | ASR (95%UI) | No.(95%UI) | EAPC_CI |
| Global |  | 0.1(0.04-0.23) | 4413(1657-9962) | 0.26(0.13-0.49) | 22601(10914) | 0.14(-0.82-1.12) |
| Sex |  |  |  |  |  |  |
|  | Female | 0.02 (0.01-0.03) | 352 (153-740) | 0.05 (0.02-0.09) | 2116 (983-4186) | 0.63 (-0.75-2.02) |
|  | Male | 0.2 (0.07-0.45) | 4060 (1487-9356) | 0.49 (0.23-0.92) | 20485 (9772-39066) | -0.48 (-1.83-0.89) |
| Age |  |  |  |  |  |  |
|  | 30-34 years | 0.11 (0.04-0.26) | 413 (154-1010) | 0.36 (0.16-0.74) | 2167 (941-4484) | 0.59 (-0.9-2.09) |
|  | 35-39 years | 0.14 (0.04-0.34) | 488 (158-1196) | 0.39 (0.17-0.84) | 2194 (937-4703) | 0.25 (-1.12-1.64) |
|  | 40-44 years | 0.13 (0.05-0.31) | 377 (130-897) | 0.45 (0.19-0.98) | 2248 (935-4902) | 0.38 (-1.13-1.91) |
|  | 45-49 years | 0.17 (0.06-0.44) | 403 (148-1013) | 0.54 (0.23-1.11) | 2578 (1085-5277) | 0.06 (-1.56-1.7) |
|  | 50-54 years | 0.2 (0.07-0.51) | 435 (158-1090) | 0.57 (0.27-1.15) | 2519 (1209-5133) | -0.07 (-1.58-1.46) |
|  | 55-59 years | 0.27 (0.1-0.68) | 505 (178-1257) | 0.67 (0.3-1.32) | 2664 (1195-5234) | -0.23 (-1.52-1.06) |
|  | 60-64 years | 0.3 (0.1-0.73) | 484 (155-1168) | 0.7 (0.27-1.51) | 2251 (869-4845) | -0.78 (-2.04-0.5) |
|  | 65-69 years | 0.44 (0.13-1.09) | 540 (159-1349) | 0.85 (0.33-1.86) | 2355 (917-5124) | -1.4 (-2.59--0.2) |
|  | 70-74 years | 0.54 (0.16-1.36) | 459 (132-1155) | 0.93 (0.33-2.13) | 1921 (685-4390) | -1.49 (-2.61--0.35) |
|  | 75-79 years | 0.34 (0.11-0.85) | 211 (68-521) | 0.75 (0.27-1.67) | 988 (360-2202) | -1.07 (-2.35-0.23) |
|  | 80-84 years | 0.2 (0.07-0.48) | 72 (26-171) | 0.53 (0.21-1.13) | 467 (184-992) | -0.33 (-1.77-1.12) |
|  | 85-89 years | 0.14 (0.06-0.31) | 21 (9-47) | 0.41 (0.17-0.86) | 188 (78-395) | 0.1 (-1.37-1.6) |
|  | 90-94 years | 0.09 (0.04-0.19) | 4 (2-8) | 0.29 (0.12-0.61) | 52 (21-109) | 0.44 (-1.16-2.06) |
|  | 95+ years | 0.06 (0.03-0.11) | 1 (0-1) | 0.18 (0.07-0.35) | 10 (4-19) | 0.79 (-1.03-2.64) |
| SDI |  |  |  |  |  |  |
|  | Low | 0.05 (0.02-0.11) | 134 (55-292) | 0.4 (0.17-0.83) | 2503 (1038-5197) | 3.5 (1.79-5.24) |
|  | Low‐middle | 0.03 (0.01-0.07) | 184 (62-507) | 0.55 (0.18-1.31) | 8872 (2914-21197) | 5.07 (2.81-7.38) |
|  | Middle | 0.2 (0.06-0.55) | 2384 (666-6401) | 0.22 (0.09-0.48) | 6238 (2663-13411) | -3.2 (-4.35--2.04) |
|  | High‐middle | 0.14 (0.04-0.34) | 1455 (449-3597) | 0.26 (0.13-0.54) | 4753 (2353-9986) | -1.19 (-2.82-0.46) |
|  | High | 0.02 (0.01-0.04) | 254 (135-465) | 0.01 (0.01-0.03) | 229 (100-517) | -4.66 (-5.65--3.67) |
| GBD regions |  |  |  |  |  |  |
|  | Oceania | 0.01(0-0.04) | 0(0-1) | 0.53(0.12-1.29) | 56(12-138) | 11.36(10.27-12.46) |
|  | Southeast Asia | 0.06(0.02-0.15) | 171(61-420) | 0.25(0.12-0.46) | 1782(825-3289) | 1.04(-0.13-2.23) |
|  | East Asia | 0.35(0.09-0.95) | 3466(895-9260) | 0.2(0.04-0.67) | 4352(841-14654) | -4.69(-5.57—3.81) |
|  | Central Europe | 0.02(0.01-0.05) | 33(14-70) | 0.02(0.01-0.05) | 36(16-76) | -2(-2.84—1.15) |
|  | Central Asia | 0.02(0-0.05) | 9(3-26) | 0.69(0.37-1.18) | 696(372-1187) | 7.84(5.51-10.22) |
|  | Eastern Europe | 0.09(0.04-0.23) | 245(106-604) | 0.97(0.51-1.65) | 2679(1399-4549) | 4.12(2.32-5.96) |
|  | Australasia | 0(0-0) | 0(0-1) | 0(0-0.01) | 1(0-3) | 1.99(1.54-2.45) |
|  | High-income Asia Pacific | 0.02(0.01-0.05) | 37(11-94) | 0.01(0-0.02) | 20(5-60) | -7.17(-8.36—5.97) |
|  | Southern Latin America | 0.01(0-0.02) | 3(1-9) | 0.01(0-0.02) | 6(1-18) | -1.16(-2.24—0.06) |
|  | Western Europe | 0.01(0-0.01) | 37(18-63) | 0(0-0.01) | 31(16-55) | -2.21(-2.95—1.48) |
|  | High-income North America | 0.01(0.01-0.03) | 47(21-86) | 0(0-0) | 9(3-24) | -6.81(-7.57—6.04) |
|  | Caribbean | 0.01(0-0.03) | 4(1-8) | 0.01(0-0.02) | 3(1-8) | -4.15(-4.8—3.51) |
|  | Andean Latin America | 0.03(0.01-0.08) | 8(2-21) | 0.1(0.04-0.22) | 64(26-143) | 0.45(-0.74-1.65) |
|  | Tropical Latin America | 0(0-0.01) | 2(0-9) | 0.03(0.01-0.1) | 90(18-267) | 4.65(2.66-6.67) |
|  | Central Latin America | 0(0-0.01) | 3(1-8) | 0.02(0.01-0.04) | 52(18-116) | 1.38(-0.1-2.88) |
|  | South Asia | 0.03(0.01-0.1) | 217(51-703) | 0.67(0.19-1.63) | 11114(3030-27313) | 5.92(4.2-7.66) |
|  | North Africa and Middle East | 0.01(0-0.01) | 12(5-23) | 0.03(0.02-0.06) | 184(93-336) | 2.18(0.53-3.85) |
|  | Central Sub-Saharan Africa | 0.06(0.01-0.19) | 18(4-54) | 0.31(0.09-0.74) | 250(70-606) | 2.9(1.82-3.99) |
|  | Western Sub-Saharan Africa | 0.04(0.02-0.07) | 38(16-75) | 0.14(0.06-0.26) | 360(163-706) | 1.51(0.2-2.84) |
|  | Eastern Sub-Saharan Africa | 0.02(0.01-0.04) | 15(6-34) | 0.27(0.14-0.49) | 600(295-1155) | 4.69(2.96-6.45) |

YLDs indicates Years Lived with Disability; EAPC, estimated annual percentage change; MDR-TB, Multidrug-resistant tuberculosis; and UI, uncertainty interval.

Table S2 The YLLs Burden of MDR-TB Attributable to Smoking in 1990 and 2021 and the Temporal Trends From 1990 to 2021

| Characteristics | | 1990 | | 2021 | | EAPC_CI |
| --- | --- | --- | --- | --- | --- | --- |
|  |  | ASR (95%UI) | No.(95%UI) | ASR (95%UI) | No.(95%UI) |  |
| Global |  | 2.64(0.9-6.74) | 114648(39268-292523) | 5.52(2.11-11.33) | 476821(182508-978954) | 0.06(-1.05-1.19) |
| Sex |  |  |  |  |  |  |
|  | Female | 0.4 (0.14-1) | 8759 (3004-21960) | 0.94 (0.31-2.08) | 41679 (13857-93133) | 0.38 (-1.09-1.87) |
|  | Male | 5.03 (1.71-12.91) | 105889 (36046-272249) | 10.29 (3.98-21.01) | 435143 (169066-887423) | -0.52(-2.08-1.08) |
| Age |  |  |  |  |  |  |
|  | 30-34 years | 2.46 (0.89-6.17) | 9478 (3413-23767) | 6.71 (2.67-13.58) | 40561 (16141-82076) | 0.33 (-1.54-2.24) |
|  | 35-39 years | 3.3 (1.14-8.49) | 11617 (4012-29910) | 8.53 (3.3-18.64) | 47825 (18511-104559) | 0.38 (-1.28-2.06) |
|  | 40-44 years | 4.14 (1.41-10.67) | 11852 (4036-30565) | 11.15 (4.15-22.26) | 55758 (20775-111366) | 0.19 (-1.51-1.91) |
|  | 45-49 years | 5.2 (1.82-13.12) | 12081 (4215-30474) | 12.93 (5.21-26.28) | 61207 (24648-124448) | -0.19 (-2.05-1.7) |
|  | 50-54 years | 6.66 (2.33-17.14) | 14150 (4949-36441) | 13.99 (5.38-29.12) | 62223 (23948-129580) | -0.35(-2.03-1.37) |
|  | 55-59 years | 7.86 (2.72-20.07) | 14564 (5043-37177) | 15.58 (5.9-33.85) | 61668 (23349-133964) | -0.35 (-1.78-1.1) |
|  | 60-64 years | 8.6 (2.83-21.38) | 13818 (4546-34342) | 16.37 (5.87-34.7) | 52391 (18799-111072) | -0.81(-2.26-0.65) |
|  | 65-69 years | 9.67 (3.09-25.21) | 11948 (3817-31166) | 14.15 (4.9-30.81) | 39040 (13530-84983) | -1.51(-2.86-0.14) |
|  | 70-74 years | 9.75 (3.1-25.28) | 8257 (2626-21399) | 13.02 (4.4-28.73) | 26802 (9056-59145) | -1.56 (-2.79--0.3) |
|  | 75-79 years | 7.24 (2.38-18.85) | 4454 (1463-11602) | 11.85 (3.89-26.4) | 15632 (5126-34819) | -1.41(-2.68-0.12) |
|  | 80-84 years | 4.97 (1.7-12.37) | 1757 (601-4375) | 9.92 (3.08-23.41) | 8685 (2701-20501) | -0.62(-2.01-0.79) |
|  | 85-89 years | 3.67 (1.29-9.14) | 555 (195-1381) | 8.02 (2.56-18.19) | 3665 (1169-8315) | -0.46(-1.83-0.93) |
|  | 90-94 years | 2.3 (0.9-5.21) | 99 (39-223) | 6.38 (2.01-14.24) | 1142 (359-2548) | -0.08(-1.47-1.33) |
|  | 95+ years | 1.9 (0.78-4.1) | 19 (8-42) | 4.07 (1.38-8.47) | 222 (75-462) | -0.98(-2.39-0.46) |
| SDI |  |  |  |  |  |  |
|  | Low | 2.09 (0.65-5.15) | 5766 (1811-14230) | 12.82 (4.67-27.57) | 81850 (30737-175025) | 2.69 (0.68-4.75) |
|  | Low‐middle | 1.24 (0.33-3.74) | 9089 (2439-27483) | 15.57 (4.47-36.66) | 254721 (73304-597358) | 4.25 (1.69-6.88) |
|  | Middle | 5 (1.34-13.91) | 60231 (16196-167002) | 3.03 (1.12-6.36) | 85555 (31690-179943) | -4.36(-5.52-3.18) |
|  | High‐middle | 3.18 (0.85-9) | 33313 (8915-94416) | 2.93 (1.61-4.83) | 52268 (28640-86557) | -3.08(-5.08-1.04) |
|  | High | 0.6 (0.26-1.24) | 6216 (2733-12845) | 0.14 (0.06-0.31) | 2283 (884-5006) | -7.26(-8.24-6.28) |
| GBD regions |  |  |  |  |  |  |
|  | Oceania | 0.32(0.06-1.21) | 13(2-48) | 10.86(2.26-28.08) | 1161(244-3058) | 10.69(9.46-11.94) |
|  | Southeast Asia | 4.52(1.02-13.17) | 2605(567-7637) | 21.13(7.49-42.17) | 26698(9473-53913) | 2.89(1.54-4.25) |
|  | East Asia | 8.15(1.91-22.59) | 80865(18834-224357) | 1.44(0.35-3.87) | 30828(7522-82820) | -7.94(-8.74—7.13) |
|  | Central Europe | 0.66(0.21-1.5) | 949(299-2144) | 0.36(0.12-0.78) | 580(190-1248) | -4.35(-5.36—3.32) |
|  | Central Asia | 0.45(0.11-1.39) | 241(60-749) | 10.57(5.62-17.19) | 10649(5666-17392) | 4.87(1.98-7.84) |
|  | Eastern Europe | 1.96(0.61-5.51) | 5220(1630-14692) | 13.36(7.33-19.83) | 37327(20480-55510) | 2.21(-0.05-4.52) |
|  | Australasia | 0.04(0.01-0.12) | 9(2-27) | 0.02(0.01-0.06) | 11(3-28) | -1.24(-1.8—0.69) |
|  | High-income Asia Pacific | 0.72(0.17-2.31) | 1474(351-4719) | 0.08(0.02-0.28) | 323(63-1047) | -9.23(-10.91—8.53) |
|  | Southern Latin America | 0.33(0.08-1.05) | 156(38-490) | 0.22(0.04-0.67) | 172(34-527) | -3.55(-4.46—2.62) |
|  | Western Europe | 0.18(0.07-0.36) | 908(336-1854) | 0.5(0.02-0.11) | 381(152-786) | -4.75(-5.41—4.09) |
|  | High-income North America | 0.38(0.14-0.82) | 1211(459-2652) | 0.02(0.01-0.07) | 129(34-359) | -8.45(-9.43—7.46) |
|  | Caribbean | 0.39(0.11-1.16) | 111(30-329) | 0.06(0.03-0.46) | 81(17-240) | -5.23(-5.94—4.52) |
|  | Andean Latin America | 2.16(0.48-6.2) | 538(121-1532) | 2.54(0.86-5.59) | 1629(551-3571) | -2.38(-3.44—1.32) |
|  | Tropical Latin America | 0.07(0.01-0.31) | 85(10-356) | 0.17(0.13-2.22) | 1879(358-5895) | 3.18(1.13-5.27) |
|  | Central Latin America | 0.15(0.04-0.38) | 147(44-383) | 0.43(0.13-0.99) | 1138(355-2611) | -0.5(-1.99-1.01) |
|  | South Asia | 2.44(0.64-7.26) | 7176(1893-22116) | 4.68(1.62-9.8) | 33708(11592-70056) | -1.13(-2.49-0.25) |
|  | North Africa and Middle East | 0.34(0.1-0.89) | 675(205-1772) | 0.91(0.31-2.09) | 5247(1735-12430) | 1.21(-0.39-2.83) |
|  | Central Sub-Saharan Africa | 2.88(0.58-9.62) | 860(172-2895) | 11.08(2.61-34.35) | 9026(2124-28306) | 1.77(0.62-2.93) |
|  | Western Sub-Saharan Africa | 1.34(0.43-3.17) | 1463(465-3496) | 4.05(1.4-8.83) | 10819(3728-23553) | 0.46(-0.93-1.87) |
|  | Eastern Sub-Saharan Africa | 0.95(0.31-2.62) | 888(286-2426) | 14(4.9-29.2) | 32847(11496-68581) | 4.58(2.6-6.6) |

YLLs indicates Years of Life Lost; EAPC, estimated annual percentage change; MDR-TB, Multidrug-resistant tuberculosis; and UI, uncertainty interval.
